# Increased Incidence of Melioidosis in Far North Queensland, Queensland, Australia, 1998–2019

**DOI:** 10.3201/eid2712.211302

**Published:** 2021-12

**Authors:** Simon Smith, Peter Horne, Sally Rubenach, Richard Gair, James Stewart, Lee Fairhead, Josh Hanson

**Affiliations:** Cairns Hospital, Cairns, Queensland, Australia (S. Smith, J. Stewart, L. Fairhead, J. Hanson);; Tropical Public Health Services, Cairns (P. Horne, S. Rubenach, R. Gair);; University of New South Wales, Sydney, New South Wales, Australia (J. Hanson)

**Keywords:** melioidosis, Tropical medicine, weather, climate, bacteria, Burkholderia pseudomallei, Far North Queensland, Australia

## Abstract

During January 1998–December 2019, the annual incidence of melioidosis in Far North Queensland, Queensland, Australia, more than doubled. Because climate and prevalence of predisposing medical conditions remained stable during that time, we hypothesize that the increased incidence was caused by urban expansion and increased construction, resulting in greater exposure to *Burkholderia pseudomallei*.

*Burkholderia pseudomallei*, an environmental gram-negative bacterium, causes the disease melioidosis. Although infection is frequently asymptomatic, melioidosis may be rapidly fatal for patients with underlying conditions that increase the risk for invasive disease. Modeling suggests that *B. pseudomallei* is ubiquitous in the tropics and that the global burden of disease is expected to rise (*1*). Indeed, increased melioidosis incidence has been documented in some countries (*2*). Although this increase may be associated with improved diagnostic capacity, it may also be explained by a growing burden of predisposing concurrent medical conditions or by greater *B. pseudomallei* exposure from environmental disruption (*3*,*4*). Changing weather patterns also have the potential to increase melioidosis incidence (*5*).

*B. pseudomallei* is endemic to Far North Queensland (FNQ), a region in the northernmost part of the state of Queensland, Australia (Figure 1). Incidence of melioidosis in the Torres Strait Islands in the region’s north is among the highest reported in published series of melioidosis cases in Australia (*4*,*6*). During the past 20 years, the FNQ population has grown rapidly, predominantly in the city of Cairns, the region’s major industrial hub, and in the nearby towns (Cairns area, in and around Cairns). This growth has necessitated substantial expansion of local infrastructure, including 2-phase development of a large motorway on the city’s southern outskirts during 2011–2017. Surveillance data suggest that this development coincided with a marked increase in the local incidence of melioidosis, primarily in the Cairns area. We aimed to determine if there was any temporospatial association between the motorway construction and the increasing incidence of melioidosis in the region or if there were other possible explanations for any observed change, with a particular focus in the Cairns area.

**Figure 1:**
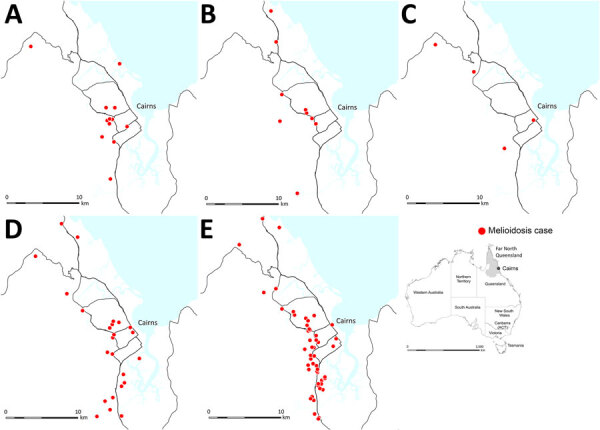
Cases of melioidosis in the Cairns area, Far North Queensland, Queensland, Australia, January 1998–December 2019. A) 1998–2002; B) 2003–2007; C) 2008–2011; D) 2012–2015; E) 2016–2019. Map shows location of Far North Queensland.

## The Study

Cairns Hospital is the sole public microbiological service provider for FNQ, a region of >380,000 km^2^ (*7*). We reviewed all culture-confirmed cases of *B. pseudomallei* infection identified in the hospital’s laboratory during January 1, 1998–December 31, 2019. Clinical details of each case were recorded as described by J.D. Stewart et al. (*4*); predisposing conditions were diabetes mellitus (glycated hemoglobin ≥6.5%), hazardous alcohol use, chronic lung disease, chronic renal disease, and immunosuppression. We used data from the Australian Bureau of Statistics to calculate disease incidence and from the Australian Bureau of Meteorology to record local climatic factors including rainfall, temperatures, cloud cover, dew points, and cyclones. For our analyses we used Stata version 14.2 statistical software (https://www.stata.com) and determined trends over time by using an extension of the Wilcoxon rank-sum test and using year of presentation as a continuous variable (*8*). We constructed maps by using MapInfo Pro 2019 Geographic Information System software (https://support.precisely.com); in the absence of a clear occupational or recreational exposure, we used participants’ residential addresses as the site of *B. pseudomallei* exposure. The study was approved by the Far North Queensland Human Research Ethics Committee (HREC/15/QCH/46-977).

A total of 297 cases of melioidosis were diagnosed during the study period, of which 284 were acquired from FNQ and included in our analysis. The mean annual incidence in FNQ increased from 4.0 (95% CI 2.7–5.2) cases/100,000 population during 1998–2002 to 9.9 (95% CI 4.9–14.9) cases/100,000 population during 2016–2019 (p<0.001) (Table 1). In the Cairns area, incidence rose from 0.6 (95% CI 0.1–1.1) cases/100,000 population during 1998–2002 to 6.6 (95% CI 3.0–10.2) cases/100,000 population during 2016–2019 (p<0.001) (Table 2; Figure 1).

**Table 1:** Incidence, predisposing conditions, and outcomes of locally acquired melioidosis cases in Far North Queensland, Queensland, Australia, January 1998–December 2019

Variable	1998–2002	2003–2007	2008–2011	2012–2015	2016–2019	p value*
Far North Queensland population, mean	220,814	232,598	256,852	272,055	283,178	<0.001
No. cases	44	41	31	56	112	<0.001
Annual incidence, cases/100,000 population, mean (95% CI)	4.0 (2.7–5.2)	3.5 (1.8–5.2)	3.0 (0–6.2)	5.1 (0.6–9.7)	9.9 (4.9–14.9)	<0.001
Age, y, median (interquartile range)	46 (32–58)	52 (40–63)	51 (38–62)	49 (42–64)	55 (47–65)	0.001
Predisposing condition, %						
Any†	73	85	84	82	90	0.02
Diabetes mellitus	50	56	58	44	58	0.59
Hazardous alcohol use	34	39	45	46	31	0.60
Chronic lung disease	7	12	16	14	16	0.13
Chronic kidney disease	16	15	3	23	17	0.48
Immunosuppression	7	12	16	20	13	0.21
Bacteremia, %	70	68	77	77	68	0.85
Case-fatality rate, %	27	15	3	11	9	0.004

*p value for trend calculated by using annual data with year as a continuous variable. †Incomplete access to patient charts from early in the study period is likely to lead to overestimation of the proportion of cases with no predisposing factor.

**Table 2:** Incidence, predisposing conditions, and outcomes of melioidosis cases near Cairns, Queensland, Australia, January 1998–December 2019

Variable	1998–2002	2003–2007	2008–2011	2012–2015	2016–2019	p value*
Cairns area population, mean	200,351	206,342	228,504	243,389	253,841	<0.001
No. cases	6	9	4	25	67	<0.001
Annual incidence, cases/100,000 population, mean (95% CI)	0.6 (0.1–1.1)	0.9 (0.1–1.6)	0.4 (0–0.9)	2.5 (0–5.8)	6.6 (3.0–10.2)	<0.001
Age, y, median (interquartile range)	45 (30–62)	65 (55–69)	49 (39–58)	56 (43–66)	56 (49–66)	0.56
Predisposing condition, %						
Any†	100	89	75	84	87	0.38
Diabetes mellitus	67	44	50	40	47	0.69
Hazardous alcohol use	67	22	75	40	28	0.046
Chronic lung disease	0	22	25	24	18	0.86
Chronic kidney disease	17	22	0	32	18	0.99
Immunosuppression	33	44	0	24	19	0.25
Bacteremia, %	67	100	100	84	72	0.18
Case-fatality rate, %	0	33	0	8	10	0.60

*p value for trend value calculated using annual data with year as a continuous variable †Limited access to charts is likely to result in incomplete documentation of risk factors from early in the study period.

During the study period, the proportion of patients in FNQ with different predisposing conditions for melioidosis did not change. The proportion of bacteremic patients also remained stable (Table 1). The case-fatality rate declined during the study period (Table 1).

In the Cairns area, where increased incidence was more marked, the small increases in mean temperature, cloud cover, and dew points in the final period of the study did not reach statistical significance. During the study period, 14 cyclones came within 200 km of Cairns, but only 1 occurred during 2016–2019 (p = 0.86) (Appendix).

**Figure 2:**
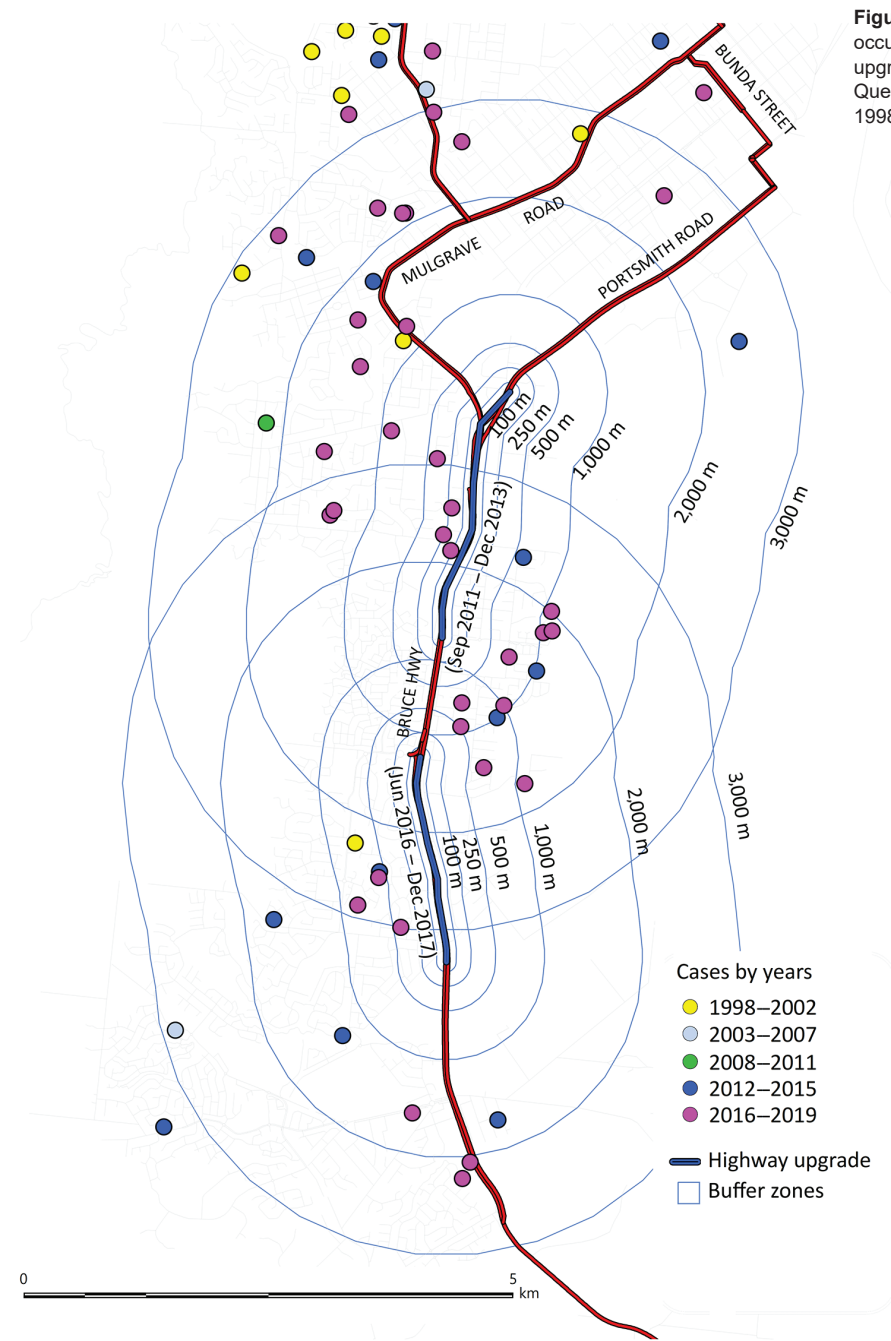
Cases of melioidosis occurring near to a motorway upgrade in southern Cairns, Far North Queensland, Queensland, Australia, 1998–2019.

Of the 284 cases included in the study, 111 (39%) were in the Cairns area; only 3 of these patients reported having an occupation in construction. Before commencement of the southern motorway expansion in the Cairns area in 2011, only 1/19 (5%) cases in the Cairns area were within 1,000 m of the existing road and 2/19 (11%) were within 2,000 m. However, after January 2012, a total of 92/168 (55%) cases occurred in the Cairns area, of which 15/92 (16%) were within 1,000 m of the highway construction and 27/92 (29%) within 2,000 m (Figure 2).

## Conclusions

The incidence of melioidosis in FNQ is rising, increasing during the study period by ≈10-fold in the Cairns area. The proportion of bacteremic patients has not changed, suggesting improved diagnosis. Similarly, we found no statistically significant change in climate or frequency of cyclones. The proportion of patients who had the common predisposing conditions remained similar. However, urban expansion may be contributing because almost a third of cases in the past 8 years of the study period occurred within 2,000 m of development of a large motorway. Of note, the motorway is built predominantly through alluvial plain soils with moderate clay content and poor drainage, which favor *B. pseudomallei* growth (*9*).

Increased rainfall, dew points, cloud cover, and temperatures have been associated with increased melioidosis cases; however, these climatic factors were stable over our study period (*5*). Cyclones have been linked to increased melioidosis cases; however, we did not observe that association in FNQ (*10*). Indeed, since 2015 when melioidosis incidence in the Cairns area sharply increased, there has been only 1 cyclone within 200 km of the area.

Why the rates of bacteremic melioidosis remain higher in FNQ than in other parts of Australia remains unclear (*11*). The higher rates may be partly explained by fewer diagnoses of cutaneous disease in rural and remote communities; however, skin swab samples are frequently taken to identify other pathogens, and skin and soft tissue *B. pseudomallei* infections are uncommon in urban areas, where most new cases have been identified (*12*). Virulence factors in local *B. pseudomallei* strains may contribute (*13*). Despite the increasing incidence, the overall case-fatality rate from melioidosis in FNQ decreased significantly during the study period, which can probably be explained by early recognition and prompt access to multimodal intensive care unit support.

Among the limitations of our study, data collection was predominantly retrospective; in addition, in the absence of clear inoculation with *B. pseudomallei*, we assumed residential addresses to be the sites of exposure. Domestic gardens are a source of melioidosis in Australia, but it is possible that unrecorded patient movements may have resulted in exposure to *B. pseudomallei* elsewhere (*14*). Additional confounding factors that increase the risk for melioidosis (e.g., socioeconomic disadvantage) may help explain regional variations in incidence, although the local geographic distribution of this socioeconomic disadvantage has not substantially changed in the past 20 years (*15*). In conclusion, although host factors and climate continue to influence the risk of acquiring melioidosis, we hypothesize that urban expansion and construction in soils harboring *B. pseudomallei* may explain the recent rapid increase in Far North Queensland, Australia. 

Appendix Supplemental results from study of increased incidence of melioidosis in Far North Queensland, Queensland, Australia, 1998–2019.
