# Supplementary material for: Increased Incidence of Melioidosis in Far North Queensland, Queensland, Australia, 1998–2019
Source: Emerg Infect Dis. 2021 Dec;27(12):3119–23. doi: 10.3201/eid2712.211302 (PMC8632158; doi:10.3201/eid2712.211302)
Supplement: Appendix — Supplemental results from study of increased incidence of melioidosis in Far North Queensland, Queensland, Australia, 1998–2019. [file 21-1302-Techapp-s1.pdf]

Article DOI: https://doi.org/10.3201/eid2712.211302

Increased Incidence of Melioidosis in Far North Queensland, Queensland, Australia, 1998–2019

Appendix

Appendix Table. Changes in climatic features in Cairns area, Far North Queensland, January 1998–December 2019

Variable	1998–2002	2003–2007	2008–2011	2012–2015	2016–2019	p*
Rainfall, mean (95% CI)	164 (103–224)	160 (104–217)	202 (129–275)	146 (94–198)	157 (93–220)	0.61
Temperature, mean (95% CI)	29.4 (28.9–29.9)	29.2 (28.7–29.8)	29.5 (28.9–30.0)	29.5 (28.8–30.1)	29.9 (29.2–30.5)	0.32
Cloud cover, mean (95% CI)	4.6 (4.1–5.0)	4.5 (4.0–4.9)	4.7 (4.2–5.1)	3.7 (3.2–4.1)	5.2 (4.3–6.1)†	0.13
Dew point, mean (95% CI)	19.3 (18.4–20.3)	19.7 (18.9–20.5)	19.8 (18.9–20.7)	18.9 (17.9–19.8)	20.1 (19.1–21.0)	0.93
No. cyclones (within 200 km of Cairns)	3	2	4	4	1	0.86

*p for trend value calculated using annual data with year as a continuous variable.
†Some measurements of cloud cover missing from Cairns Aero Station located ≈7km from the center of Cairns City and taken from Cairns Racecourse Station located ≈4km from the center of Cairns City.
